# Digital Image Analysis Assisted Microradiography—Measurement of Mineral Content of Caries Lesions in Teeth

**DOI:** 10.6028/jres.096.009

**Published:** 1991

**Authors:** L. C. Chow, S. Takagi, W. Tung, T. H. Jordan

**Affiliations:** American Dental Association Health Foundation, Paffenbarger Research Center, National Institute of Standards and Technology, Gaithersburg, MD 20899

**Keywords:** demineralization, dental caries, digital image analysis, microradiography, remineralization, tooth mineral

## Abstract

This study investigated the feasibility of using a digital image analysis system to process the information contained in microradiographs of tooth sections that included dental caries lesions. The results show that by using an aluminum step wedge to provide a range of thickness standards and a sound area of the sample as an internal reference, data on tooth mineral content as a function of the location can be obtained with an estimated error of less than 5% relative to the mineral content of sound area. This microradiographic technique allows the response of tooth samples to a remineralization treatment to be quantitatively measured and statistically analyzed.

## 1. Introduction

Contact microradiography is an important and frequently used technique [1–4] for qualitative assessment of the mineral content of partially demineralized lesions in tooth enamel or root dentin. With a scanning microdensitometer connected to a computer, quantitative information on the mineral content of different regions in the lesion can also be obtained from microradiographs [5,6]. Digital image analysis systems that are capable of transforming photographic images, including those from microradiographs, into digital forms have become widely available in recent years. The digitized microradiographs can also be used to obtain quantitative information on the mineral content distribution as a function of location in the lesion [7]. The precision and sensitivity of this type of measurement are affected by numerous factors including parameters associated with the radiographic procedure, the quality of the microscope, and the characteristics of the digital image analysis system, e.g., spatial resolution, range of grey levels (brightness), etc. The present study examined the feasibility of quantitation of the mineral content in caries lesions using a digital image analysis system capable of producing 256 grey levels. Mineral contents of the lesions in extracted human root dentin were measured before and after a remineralization treatment. Several factors associated with the radiographic procedures or with the densitometric measurements that may produce significant errors in the final results were investigated. A procedure that can produce data on mineral density distribution with an estimated error of below 5% is described.

## 2. Materials^3^ and Methods

### 2.1 Thickness Standards

**Figure 1:**
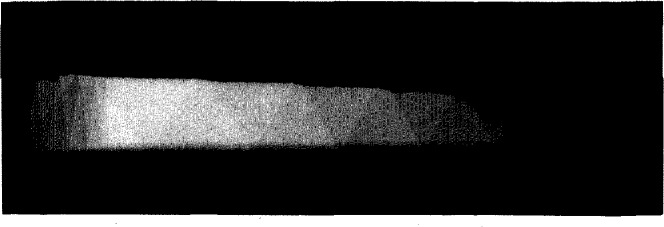
An aluminum step wedge used as the thickness standards.

As is common to all quantitative analytical procedures, it is necessary to have a set of standards of known values of the property being measured, i.e., mineral content in the present case. It can be shown [5] that under a given measurement condition, a fixed relationship exists between the radio-opacities of aluminum and enamel (or root). Thus, an aluminum step wedge was used as a set of thickness standards. These, in turn, were used as the standards for measuring the mineral content of tooth specimens. A total of four wedges were prepared by folding 15 μm thick aluminum foils (A.D. Mackay, Inc., New York, NY) such that each step in the wedge contained from 1 to 13 layers of the foil (fig. 1), yielding thicknesses ranging from 15 to 195 μm. The first eight layers of the standards were used because the radio-opacities of these standards encompassed but did not greatly exceed those of partially demineralized and intact areas of the root dentin specimens used in the present study. The overall dimension of the wedge was approximately 2 mm in width by 17 mm in length.

### 2.2 Fonnation of Caries Lesions in Tooth Specimens

A pH 4 acetate-containing solution [8] was used to form caries-like lesions in the roots of human molar teeth extracted for orthodontic reasons. The demineralized root samples were sectioned longitudinally (along the length of the root) with a diamond blade (Isomet, Buehler Ltd., Lake Bluff, IL). The sections were then ground by hand on wet 600 grit sand paper (Buehler Ltd., Lake Bluff, IL) to a thickness of approximately 120 μm. Radiographs of four root dentin sections containing lesions were produced by the procedure described below.

### 2.3 Remineralization Treatment

**Figure 2:**
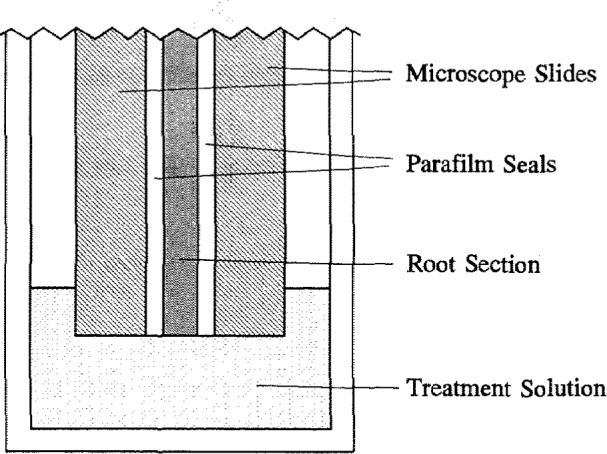
Schematic drawing of a thin section of tooth in a holder which protected the cut surfaces of the specimen from being exposed to the treatment solution.

Following documentation of the lesions on the radiographs, the specimens were treated with a remineralizing solution ([Ca] =1.5 mmol/L, [P] = 1.2 mmol/L, [F] = 53 μmol/L, [HEPES] = 25 mmol/L, [KCl] = 30 mmol/L, pH = 7.4). The solution was applied to the single section specimens, each placed in a holder consisting of two tightly held microscope glass slides and a layer of parafilm between the tooth section and each slide (fig. 2). In a previous study [9] this assembly was immersed in a pH 7 methylene blue (1 wt. %) dye solution for 5 days. No penetration of the dye into the tooth-parafilm or the parafilm-glass interface was detected, indicating the presence of water-tight seals at the interfaces. Thus when the tooth section in the holder was immersed in the remineralizing solution, only the natural surfaces of the tooth, positioned near the lower edges of the slides, would be exposed to the solution. Tooth sections were exposed to the remineralizing solution for 5 days, and the solution was changed daily. After the remineralization treatment the specimens were removed from the holders and radiographs were again produced.

### 2.4 Microradiography

Contact microradiographs of the tooth specimens were obtained with Ni-filtered Cu*K_α_* radiation (Faxitron, Model 43855A, Hewlett Packard, McMinnville, OR) operating at 40 kV and 3 mA. The x-ray source to film distance was 30.5 cm. The exposure time used was 13 min, which was determined in preliminary experiments as optimum for the type of specimens used. A fine grain film (Kodak Professional film S0343, Eastman Kodak Co., Rochester, NY) designed for contact microradiography was used. The x-ray films were developed according to the procedure recommended by the manufacturers. Radiographs containing either four aluminum step wedges or several tooth sections and one step wedge were produced.

### 2.5 Digital Image Analysis System

A commercially available digital image analysis system (Bioquant System IV, R & M Biometrics, Inc., Nashville, TN) was interfaced to a microscope (Leitz Ortholux, Germany) and a personal computer. The image analysis system consisted of a monochrome video camera (Model 65, Mk IV Series, Dage-MTI, Inc., Michigan City, IN), a pointer device, a frame grabber card capable of storing an image containing 252 × 246 pixels (picture elements) with 256 grey levels, and the necessary software for video counting and microdensitometry measurements. In this setup, the maximum spatial resolution, as determined by the optical magnification of the microscope (250X) and the number of pixels included in the image, was approximately 1.10 and 1.45 μm in the horizontal and vertical directions, respectively. After the digitized images of the standards and samples were obtained, the data were processed, as described below, using additional software developed specifically for microradiographic study on tooth specimens [10,11].

### 2.6 Precision of Radiographic Measurements

In a typical radiograph produced for measurement of tooth mineral contents, there is a distance, usually of several millimeters, between the standards and the specimen. Variations in brightness and contrast in the radiograph over such distances can contribute to errors in the measurement. An experiment consisting of the following steps was conducted to determine the extent of variation in radio-opacity of identical objects placed at different sites of a given radiograph: (1) A microradiograph containing four aluminum step wedges was obtained under the conditions outlined above. (2) The microradiograph was placed on the microscope to which the digital image analysis system was attached, and the average grey level of each step of all the wedges was measured in triplicate. (3) For each wedge the known thicknesses (*Th*_k_), in the unit of number of layers, were plotted against the measured grey level (*GL*) values, and a calibration curve was constructed by fitting the data to a first, second, or third order polynomial function (eqs (1), (2), and (3), respectively) with the least squares method.

$$Th_{k}=a_{0}+a_{1}GL$$ (1)

$$Th_{k}=a_{0}+a_{1}GL+a_{2}GL^{2}$$ (2)

$$Th_{k}=a_{0}+a_{1}GL+a_{2}GL^{2}+a_{3}GL^{3}$$ (3)

(4) Steps (1) through (3) were repeated such that data on a total of twenty different step wedge images from five radiographs were obtained. (5) For each type of regression function, the average standard error of *y* estimate and the correlation coefficient were computed to determine the goodness of fit (table 1). (6) One step wedge in each radiograph was randomly selected to serve as the standard, and the other three step wedges were treated as samples, each having segments of different thicknesses. (7) The measured grey level values for the “samples” were used to compute the corresponding calculated thicknesses by use of the calibration curve obtained from the “standard” of that radiograph. (8) A linear regression analysis with eq (4) was performed to determine the correlation between the calculated thicknesses (*Th*_c_) and the known thickness (*Th*_k_) for each aluminum wedge (table 2).

$$Th_{c}=b\;Th_{k}$$ (4)

Equation (4), which represents a linear model with forced zero intercept, was chosen for the reason given in section 4.

The conditions used for developing the radiographs and the light intensity of the microscope inevitably varied slightly from one measurement to the next. These have lead to the differences in brightness and contrast of the digitized radiographs as would generally be encountered in the measurement.

### 2.7 Assessment of Mineral Content

**Figure 3:**
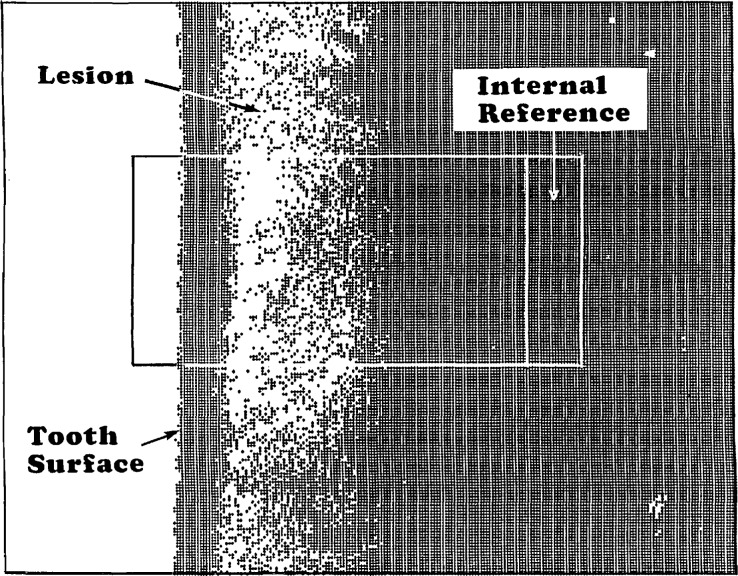
A digitized microradiographic image of a root specimen containing a lesion with a mineral dense surface layer. Black dots are pixels where the percent mineral contents were 60 or higher.

This procedure consisted of the following steps: (1) A microradiograph containing images of the tooth specimens and a step wedge was obtained. (2) The radiograph was placed under the microscope, and the average grey levels of the standards were measured in triplicate as described earlier. (3) The data were processed and a standard curve was constructed as before. (4) An image of an area of the sample approximately 277 μm high × 357 μm wide containing the lesion being studied (fig. 3) was “captured” digitally, and the grey levels of all the pixels in the image were stored in a “grey level” file in the computer. (5) The grey levels of the individual pixels stored in the file were converted to thickness values through the use of the standard curve obtained in (3), and these were stored in a “thickness” file. (6) An area, approximately 100 μm high and 25 μm wide, located in the sound portion of the specimen (fig. 3) was chosen as the internal standard reference for that sample. (7) The average thickness of all the pixels located within this internal reference area was calculated, and this value was considered to be equivalent to a mineral content of 100%. (8) The thickness values of the individual pixels of the digitized image were then divided by the average thickness of the internal standard. Each pixel now had a value in the unit of *%* mineral content relative to the sample internal standard. (9) A window covering an area 70 pixels (102 μm) high and 200 pixels (220 μm) wide of the sample including the lesion and the internal reference was delineated (fig. 3), and the mean and standard deviation of the % mineral content of all the pixels within each column, i.e., at a given distance from the root surface, were calculated. (10) The data obtained were used to produce a mineral content profile of the specimen, i.e., the average mineral content as a function of distance (from the tooth surface toward the pulp).

## 3. Results

**Figure 4:**
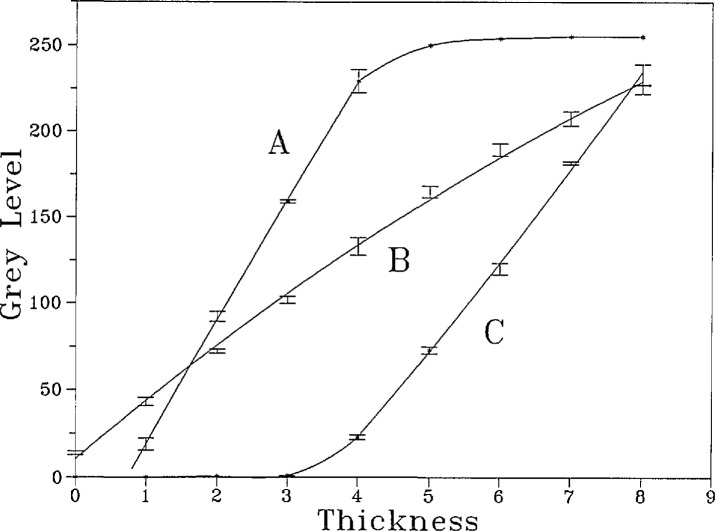
Relationships between grey level and thickness of aluminum step wedge under different radiographic or microscope illumination conditions (see text). The bars denote standard deviations (*n* =3).

Curve B in figure 4 shows the relationship between the measured grey level values and the thickness of an aluminum step wedge. Regression statistics (table 1) of the data on 20 radiographic images of the step wedges from 5 radiographs shows that both the second [eq (2)] and third [eq (3)] order polynomial functions provided a better fit of the thickness vs. grey level data than did the first order-function [eq (1)]. Since there was no statistically significant difference between the second and the third order functions, in subsequent calculations the second order function was used for constructing the calibration curves. Data in table 1 show that for the second order curve the average standard error of thickness estimate is 0.135 layers.

**Figure 5:**
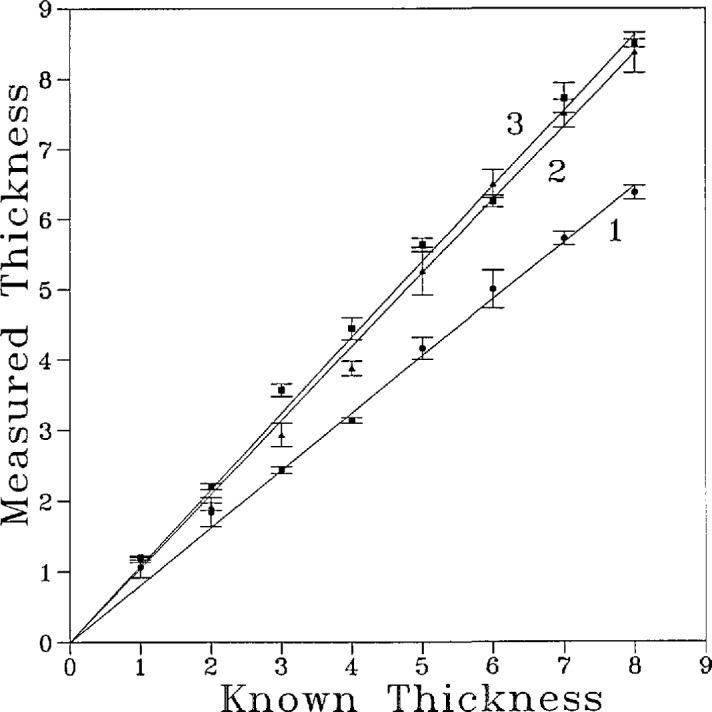
Relationships between known thicknesses and thicknesses calculated from the grey level values measured for the three step wedges in radiograph E (table 2). The straight lines are least square lines with forced zero intercept [eq (1)]. The bars denote standard deviations (*n* =3).

In figure 5 the calculated thicknesses were plotted against the known thicknesses for the three step wedges from radiograph E (table 2). There was a good linear relationship between the two parameters. Listed in table 2 are the results of linear regression analysis of the data by the zero intercept model. It is seen that the calculated thickness and actual thickness are strongly correlated (mean correlation coefficient = 0.995). However, the slopes of the straight lines ranged from 0.809 to 1.528, suggesting considerable discrepancies between the calculated and the actual thicknesses. The mean standard error of the estimated *Th*_c_ is approximately 0.2 layers (table 2).

**Figure 6:**
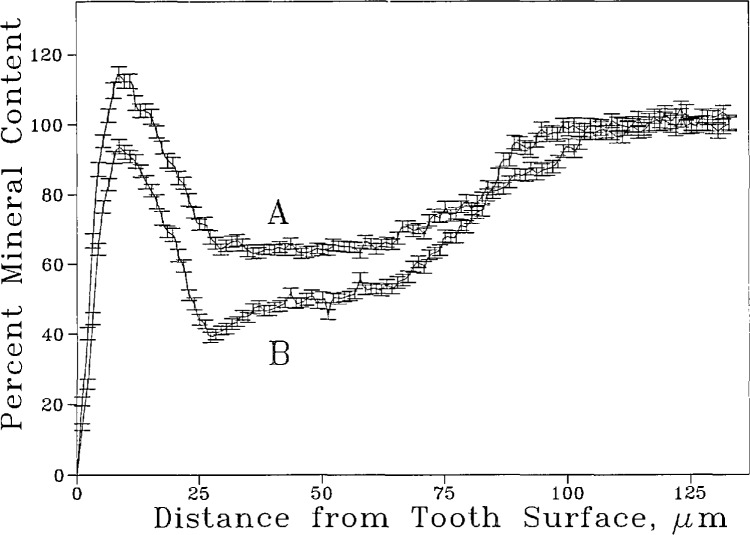
Mineral content profile of a root sample with lesion before (B) and after (A) a remineralization treatment. The profiles shown are averages of a window approximately 100 μm in height (70 pixels) as shown in figure 3. The bars denote standard errors of the mean (*n* =70).

**Figure 7:**
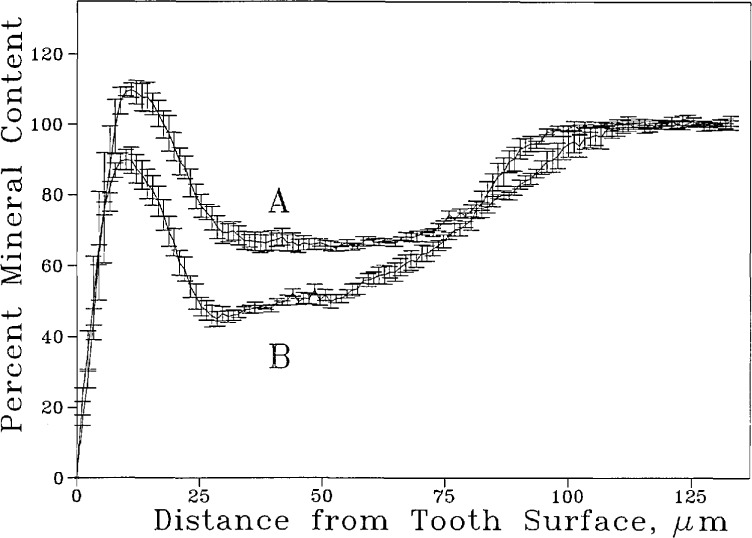
Average mineral content profile of 4 root lesions before (B) and after (A) a remineralization treatment. The bars denote standard errors of the mean (*n* =4).

Curve B in figure 6 shows the mean and standard deviation of % mineral content of a lesion as a function of distance from the tooth surface. The peak on the left corresponds to a mineral dense layer at the tooth surface preceding the body of the lesion. The mineral density profile of the same lesion after re-mineralization treatment is also shown (curve A, fig. 6). A two-tailed t test of the data shows that for this sample the increase in mineral content was significant in the area of the lesion from approximately 5 to 70 μm from the tooth surface. Figure 7 shows the average mineral profile of all four specimens before remineralization (curve B) and after the remineralization treatment (curve A). To gain a more quantitative picture, the average mineral gains in the various zones of the lesions (in 10 μm intervals from the tooth surface) were calculated from the data (table 3). The results show that immersing the root lesions in the pH 7.4 calcium phosphate solution produced a small but statistically significant increase in mineral content of the lesion to a depth approximately 70 μm.

**Figure 8:**
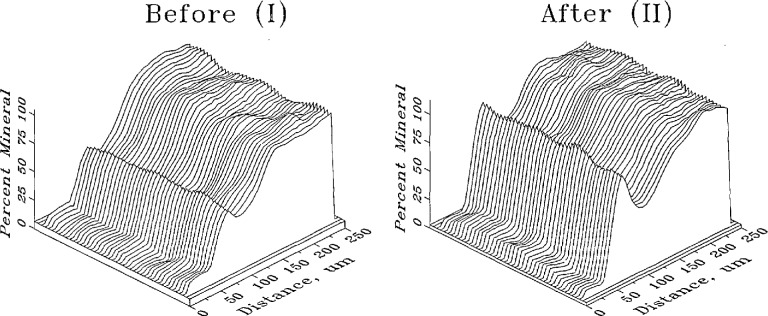
Mineral profiles of a root specimen with lesion before (I) and after (II) remineralization treatment. The horizontal axes are location coordinates and the vertical axis represents the mineral content.

**Figure 9:**
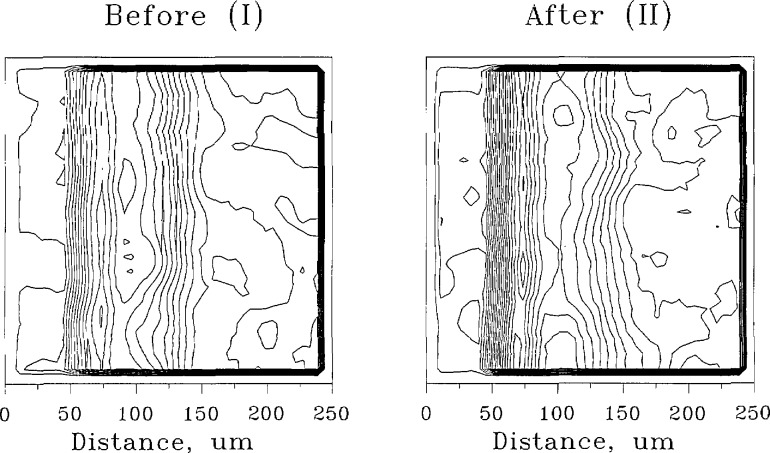
Isodensitraces of the same specimen as in figure 8 before (I) and after (II) remineralization treatment. The lines denote steps of 5% change in mineral content.

Data contained in the digitized images of a caries lesion may be used to plot mineral content profiles in a three-dimensional coordinate system with the horizontal axes representing the location coordinates and the vertical axis representing the percent mineral content. Figures 8 (I) and 8 (II) show the images of a lesion before and after the remineralization treatment, respectively. The same data may also be used to produce isodensitraces of the radiographs [figs. 9 (I) and 9 (II)] in which the contour lines correspond to steps of *5%* change in the mineral content. A commercially obtained software package (SURFER, Golden Software, Golden, CO) was used to produce the plots.

## 4. Discussion

The relationship between the grey level and the thickness of the aluminum step wedge is determined by the brightness and contrast of the radiographic image recorded in the digitized form. Figure 4 shows three characteristic relationships between the two parameters in three characteristic situations: (1) a near linear relationship holds for the lower portions of the standards, but after a certain thickness the grey level reaches the saturation values of 255 (curve A), (2) a near linear relationship prevails for the entire range of thickness (curve B), and (3) the grey levels for the first several standards were essentially zero, and a near linear relationship holds thereafter (curve C).

The brightness determines the horizontal location, whereas the contrast determines the slope of the standard curve. Thus curve A in figure 4 was obtained from an image with a greater brightness than the one from which curve C was obtained. Further, the radiographs that produced curves A and C had greater contrast than the one that produced curve B.

In the present experimental setup, the brightness and contrast of the digitized radiographic image are controllable by the following factors: (1) the brightness and contrast of the original radiograph; these, in turn, were determined by the x-ray exposure conditions and those used for developing the film, (2) light intensity of the microscope, which affects the brightness of the image, and (3) additional controls of brightness and contrast provided by the digital image analysis device through variable attenuation of the signals from the video camera.

Since curve B shown in figure 4 provides a more consistent relationship between grey level and thickness, the results reported in the present study were obtained under experimental conditions that would produce this type of calibration curve. It is noted that under some conditions curve A or C (fig. 4) may be desirable since it may allow for greater sensitivity of measurement because of the larger slope in the usable portion of the standard curve. On the other hand, since the standards are indistinguishable from one another over a portion of the curve (e.g., steps 5 through 8 for curve A), substantial errors would result should the sample mineral content fall within this region of the calibration curve.

Discrepancies in the calculated thickness and actual thickness of step wedges located in different areas of a given radiograph (fig. 5) are presumably due to variations in the contrast and brightness over the relatively small distances separating the wedges. In some cases, e.g., E1 in table 2, the error was over 20%. This suggests that errors of such magnitude may be present in the mineral content of a tooth specimen estimated by a calibration curve established from a set of standards situated just 1 or 2 mm away on the same radiograph. Fortunately, data obtained in the present study indicate that the calculated and actual thickness are linearly correlated (fig. 5 and table 2). The linear relationship, with the intercept set to zero, makes it possible to correct for the deviation if an additional internal standard located within the sample is available. In the case of measuring the mineral content of a tooth specimen with caries lesions, the sample internal standard may be obtained by assigning an area located in the sound portion of the specimen to have 100% mineral content. Thus, the mineral content of the entire sample may be recalculated as percent of the mineral content of the internal standard. The data obtained from the step wedges indicate that when this procedure is used, the standard error of the estimated thickness is approximately 0.2 layers (table 2). Since the radio-opacity of the sound portion of a typical tooth sample is comparable to that of 5 to 7 layers of aluminum, the standard error in the estimated mineral content would be in the range from 4 to 2.9% of the mineral content of the sound root dentin or enamel.

Quantitative microradiography using the procedure described here has advantages over that conducted by scanning the radiograph with a micro-densitometer [5,6]. The digital image analysis method captures from the radiograph information on the mineral content in a two-dimensional area of the specimen. The digitized radiographic image obtained is equivalent to hundreds of contiguous scannings of the microradiograph at approximately 1.5 μm intervals. The mineral content profile produced (fig. 6) reveals both the mean and the variance of the mineral content as a function of distance from the tooth surface in a specific area of the specimen. Since this method does not require mechanically moving the densitometer, it generally can produce a higher spatial resolution than that obtainable in the scanning method. Elliott et al. [12] reported a scanning x-ray microradiographic system in which the x-ray absorption by the specimen was measured directly without using photographic recordings. By step-by-step translation of the specimen, a two-dimensional radiographic image with a resolution of 20 μm and a reproducibility of 1.5% can be obtained. Compared to this system, the digital image analysis method requires substantially shorter measurement times and produces higher spatial resolution. Photographic artifacts, which are common to all methods using photographic film recordings, are minimized by the use of thickness standards and an additional sample internal reference described above.

Like many other biological samples, tooth specimens are highly variable in their response to a given treatment. Consequently, a large sample size is often needed to detect small differences in mineral content distribution resulting from a remineralization treatment. The procedure investigated in the present study allows the mean and standard deviation of mineral content distribution (as a function of distance from the tooth surface) of a designated area of the lesion to be measured both before and after certain treatment. The quantitative nature of the measurement facilitates statistical analysis of the data and aids in detection of small but significant differences such as that shown in figure 7.

## Figures and Tables

**Table 1:** Regression statistics of thickness of aluminum wedges vs grey level

Wedge	Polynomial Function					
	1st order		2nd order		3rd order	
	S.E.^a^	R^b^	S.E.	*R*	S.E.	*R*
A1	0.254	0.994	0.195	0.997	0.133	0.998
A2	0.164	0.997	0.133	0.998	0.126	0.998
A3	0.136	0.997	0.080	0.999	0.083	0.999
A4	0.233	0.996	0.187	0.998	0.192	0.998
B1	0.136	0.997	0.096	0.999	0.089	0.999
B2	0.090	0.998	0.090	0.998	0.070	0.999
B3	0.158	0.994	0.127	0.998	0.132	0.997
B4	0.111	0.996	0.048	0.999	0.034	0.999
C1	0.137	0.998	0.099	0.999	0.085	0.999
C2	0.257	0.992	0.182	0.996	0.190	0.989
C3	0.173	0.996	0.143	0.998	0.144	0.998
C4	0.190	0.994	0.074	0.999	0.074	0.999
D1	0.145	0.998	0.143	0.998	0.146	0.998
D2	0.108	0.999	0.101	0.999	0.070	0.999
D3	0.211	0.995	0.173	0.997	0.067	0.999
D4	0.180	0.992	0.064	0.999	0.056	0.999
E1	0.400	0.987	0.245	0.996	0.238	0.996
E2	0.354	0.990	0.200	0.997	0.196	0.997
E3	0.184	0.996	0.156	0.997	0.155	0.998
E4	0.441	0.985	0.160	0.998	0.164	0.998

Mean	0.200	0.995	0.135	0.998	0.122	0.998
S.D.	0.101	0.003	0.053	0.001	0.056	0.001

a S.E. = Standard error of *y* estimate.

b R = correlation coefficient.

**Table 2:** Regression statistics of measured thickness vs. known thickness of aluminum step wedges.

Radiograph	Wedge	Slope	(S.E.^a^)	Correlation coefficient	S.E. of *y* estimate
A	1	0.987	(0.008)	0.997	0.157
	2	1.294	(0.006)	0.999	0.107
	3	0.917	(0.009)	0.994	0.246
B	1	1.009	(0.019)	0.989	0.245
	2	1.063	(0.010)	0.996	0.134
	3	1.511	(0.012)	0.998	0.115
C	1	0.946	(0.010)	0.995	0.201
	2	0.987	(0.010)	0.995	0.198
	3	1.276	(0.014)	0.994	0.227
D	1	1.004	(0.007)	0.997	0.143
	2	1.086	(0.011)	0.995	0.210
	3	1.528	(0.028)	0.985	0.334
E	1	0.809	(0.006)	0.996	0.216
	2	1.046	(0.013)	0.993	0.258
	3	1.078	(0.008)	0.997	0.218

	Mean	1.103	(0.011)	0.995	0.201
	S.D.	0.202		0.003	0.059

a Standard error of the slope.

**Table 3:** Change in mineral content (%) of root dentin lesion produced by the remineralization treatment

	Distance (μm) from tooth surface							
	1–10	11–20	21–30	31–40	41–50	51–60	61–70	71–80
Before	57.5	79.5	51.0	47.2	50.8	52.7	59.0	67.8
S.D.^a^	(1.2)	(6.8)	(4.4)	(1.6)	(1.9)	(2.1)	(3.8)	(3.0)
After	67.8	103.0	78.0	67.5	66.3	65.9	67.5	72.6
S.D.	(8.2)	(5.0)	(4.5)	(4.0)	(2.8)	(1.1)	(0.5)	(1.1)
Paired								
Difference	−0.8^b^	20.9	28.5	21.4	15.7	12.9	7.5	3.3^b^
S.D.	(7.5)	(5.6)	(3.3)	(3.8)	(3.6)	(2.6)	(3.4)	(3.1)

a S.D. = standard deviation of the mean; *n* = 4.

b Statistically non-significant (*p* > 0.05).

## Footnotes

3 Certain commercial equipment, instruments, or materials are identified in this paper to specify adequately the experimental procedure. Such identification does not imply recommendation or endorsement by the National Institute of Standards and Technology or the ADA Health Foundation, nor does it imply that the materials or equipment identified are necessarily the best available for the purpose.
